# Monomeric [CRP] and CRP-Controlled Stress and Pain Hypersensitization as Novel Predictors of Cognitive Disturbance and AD in Chronic Inflammatory Disease

**DOI:** 10.3390/ijms262311279

**Published:** 2025-11-21

**Authors:** Mark Slevin, Amelia Tero-Vescan

**Affiliations:** 1 Centre for Advanced Medical and Pharmaceutical Research, George Emil Palade University of Medicine, Pharmacy, Science, and Technology of Târgu Mureș, 38th Gh. Marinescu Street, 540139 Târgu Mureş, Romania; 2 Department of Medical Chemistry and Biochemistry, Faculty of Medicine in English, George Emil Palade University of Medicine, Pharmacy, Science, and Technology of Târgu Mureș, 38th Gh. Marinescu Street, 540139 Târgu Mureş, Romania; amelia.tero-vescan@umfst.ro

**Keywords:** C-reactive protein, monomeric C-reactive protein, interleukin-6, neuropsychological, stress, inflammation, Alzheimer’s disease

## Abstract

Chronic low-grade systemic inflammation is increasingly recognized as a key mediator linking stress, pain sensitivity, and cognitive decline. Central to this process are the acute-phase reactants interleukin-6 (IL-6) and C-reactive protein (CRP), which serve as biomarkers of systemic inflammation while promoting neuroimmune dysregulation. Emerging evidence implicates the IL-6–CRP axis in the amplification of pain perception, central sensitization, and stress hypersensitivity, ultimately promoting neurodegenerative processes such as those observed in Alzheimer’s disease (AD) and vascular dementia. Monomeric CRP (mCRP), a proinflammatory isoform generated under mechanical or oxidative stress, can trigger histone modifications (e.g., H3 citrullination), activate endothelial and immune cells, and exacerbate inflammatory pain pathways. These mechanisms are further modulated by genetic and epigenetic factors, including IL-6/CRP/NR3C1 gene variant expression; promoter methylation; and stress-responsive microRNAs, which intersect with dysregulation of the hypothalamic–pituitary–adrenal (HPA) axis, impairing immune resolution and neurocognitive resilience. Psychosocial stressors, such as the burden of caregiving or perfectionistic cognitive patterns, amplify IL-6 and CRP levels, particularly when pain is present, suggesting a synergistic interaction between emotional distress and somatic inflammation. Specifically, elevated CRP is associated with increased pain sensitivity, lower pain thresholds, and cognitive decline even in subclinical populations, providing a feedforward model in which chronic stress and pain potentiate systemic inflammation, disrupt neuroendocrine feedback, and accelerate neurodegenerative pathology. However, in this model, the potentially critical mechanistic and pathological role of mCRP remains to be discovered. This review addresses the missing elements of these overlapping pathways and discusses the therapeutic potential of targeting IL-6–CRP signaling, stress regulation, and epigenetic modifiers as strategies to ameliorate inflammation-driven cognitive decline and enhance stress resistance in chronic disease contexts. We propose that plasma mCRP or more likely the isoform-aware metric, the mCRP/CRP ratio, will provide a biologically anchored, potentially more discriminative approach to vascular-neuroimmune risk and capture the propensity for local effector signaling, likely outperforming hs-CRP or IL-6 alone for risk stratification across neurovascular and stress-sensitized pain phenotypes.

## 1. Introduction

Pentameric C-reactive protein (CRP/hsCRP) and its counterpart interleukin-6 (IL-6 are key acute-phase reactants recognized as biomarkers and potential mediators at the interface between systemic autoimmune inflammation and neurodegenerative disease, particularly Alzheimer’s disease (AD). Elevated CRP levels are common in autoimmune conditions such as systemic lupus erythematosus and rheumatoid arthritis, where chronic inflammation can extend beyond peripheral tissues to impact the central nervous system (CNS) [1]. Mechanistically, CRP, especially its monomeric form (mCRP), can cross the blood–brain barrier (BBB) under pathological conditions, contributing to microglial activation, engagement of the complement cascade, and disruption of neuronal homeostasis [2]. Furthermore, longitudinal studies have demonstrated that elevated baseline CRP is associated with an increased risk of cognitive decline and AD development, potentially through sustained IL-6-mediated inflammatory signaling and vascular injury pathways [3].

Recently, Long et al. (2023) [4] performed a meta-analysis screening CRP levels associated with cognitive assessment or formal dementia diagnoses. Using a random-effects model, the authors found no significant association between high CRP levels and general cognitive decline (OR = 1.115, *p* = 0.469), even after adjusting for covariates. However, elevated CRP was significantly associated with an increased risk of progression from normal cognition to dementia (HR = 1.473, *p* = 0.0394; adjusted HR = 1.429, *p* = 0.010). Subgroup analysis revealed that higher CRP levels were correlated with deterioration in visuospatial ability and a notably greater risk of conversion to vascular dementia (OR/HR = 2.769, *p* = 0.000). These findings implicate CRP as a novel biomarker for identifying individuals at increased risk of developing dementia, particularly those of vascular origin [4].

Elevated levels of IL-6 and CRP, which are markers of systemic inflammation, have also been associated with stress hypersensitivity and cognitive disturbances. For example, in long-term survivors of Hodgkin lymphoma, higher IL-6 concentrations are correlated with poorer visuomotor processing speed, whereas elevated high-sensitivity CRP (hs-CRP) levels are linked to deficits in attention, memory, and executive function, providing more evidence for a role for systemic inflammation in neurocognitive dysfunction [5]. Similarly, in patients with severe craniocerebral injuries, increased IL-6 and CRP levels are associated with increased oxidative stress and greater cognitive impairment, indicating that their association with inflammation through mitochondrial activation and ROS may also exacerbate neurological damage [6]. Furthermore, genetic variations in the CRP gene have also been linked to higher CRP levels, more severe symptoms, and poorer cognitive function in civilian women with posttraumatic stress disorder (PTSD), highlighting the strong associations among inflammation, stress-related disorders, and cognitive decline [7].

In this review, we explore the role of CRP and IL-6 not only as biomarkers of systemic inflammation but also as active participants in the pathophysiology of neurodegenerative and stress-related disorders. These acute-phase reactants are increasingly implicated in mediating the interface between chronic inflammation, heightened pain and stress sensitivity, and cognitive dysfunction. While CRP, particularly its monomeric form, can cross the BBB and activate microglia, IL-6 is a key driver of downstream inflammatory signaling, which is implicated in neurovascular damage and neuronal dysfunction. Importantly, these inflammatory mediators not only reflect disease activity but also exacerbate neuroinflammation and neurodegeneration, potentially accelerating the onset of conditions such as AD [8]. This review considers current evidence supporting a mechanistic link between mCRP/CRP/IL-6 signaling and the amplification of stress and pain sensitivity, contributing to a broader understanding of how systemic inflammation may drive vulnerability to neurodegenerative disease. As an additional hypothesis, we investigated whether heightened sensitivity to chronic or repeated acute bouts of pain could elicit an exaggerated IL-6-CRP axis-driven neurodegenerative cascade leading to cognitive disturbance and AD, highlighting the critical biomarkers of hypersensitivity that could stratify and be used in prognostic distinctions and risk analysis.

In interpreting CRP biology, context is everything: the individual’s current physiology (acute vs. chronic inflammation), vascular surface activation, and comorbid conditions shape how much circulating pentameric pCRP is converted into mCRP. Although higher pCRP often coincides with higher plasma mCRP, the relationship is variable because mCRP is generated locally on activated membranes (endothelium, platelets, and microvesicles) and much of it remains -associated with tissue or particles. Consequently, mCRP levels are frequently distinct from total CRP levels and can carry independent prognostic information, supporting its use as a stand-alone biomarker/predictor rather than merely a derivative of hs-CRP. For vascular and neuroinflammatory risk stratification, measuring both absolute mCRP and the mCRP/CRP ratio can better capture the underlying inflammatory context than can hs-CRP alone.

NOTE: In most published studies, no clear discrimination was made between circulating CRP and mCRP, largely because the specific antibodies required to distinguish mCRP from native pentameric CRP (pCRP) were not yet available. Therefore, interpretations of CRP-associated pathology must be made with caution, as the relative contribution of each isoform to diagnostic or prognostic outcomes remains uncertain. Where mCRP was specifically quantified, it is clearly designated as such in the present text [9,10].

## 2. IL-6 and C-Reactive Protein as Central Mediators in Chronic Inflammation and Neuropsychological Disorders

Chronic inflammation is increasingly recognized as a key ‘biological substrate’ linking a diverse array of systemic diseases with psychiatric comorbidities, notably depression and anxiety. Among the inflammatory mediators, IL-6 and CRP have consistently shown strong correlations with disease severity and neuropsychiatric symptoms, acting as both biomarkers and possible contributors to disease pathogenesis. Several large-scale cohort and genetic studies have substantiated this link. Milaneschi et al. (2021) [11] analyzed data from over 150,000 participants in the UK Biobank and NESDA studies and used the serum concentrations of CRP and IL-6 along with Mendelian randomization (MR) to examine causal relationships. They reported that higher CRP levels were significantly associated with depressive symptoms such as fatigue (OR = 1.12), depressed mood (OR = 1.06), and sleep problems (OR = 1.05). Elevated IL-6 levels were similarly associated with anhedonia (OR = 1.30), sleep disturbances, and appetite changes, suggesting a neurovegetative profile of inflammation-related depression. MR analyses also indicated a potentially causal role for IL-6 signaling in depression [11].

Beurel et al. (2020) [12] provided mechanistic insight into how chronic inflammation can exacerbate mood disorders. Their review highlighted that both innate and adaptive immune dysregulation in major depressive disorder (MDD) lead to persistent elevation of CRP and IL-6, promoting microglial activation and impaired synaptic plasticity, which hypothetically could hinder the response to antidepressants [12]. In terms of methodology, these findings are supported by a combination of prospective cohort designs, multivariate regression analyses adjusting for key covariates (e.g., age, sex, body mass index (BMI), smoking), and biological assays (e.g., ELISA for IL-6 and high-sensitivity CRP measurement). Additionally, meta-analytic data reviewed by Irwin et al. (2016) [13] involving over 50,000 individuals demonstrated that both sleep disturbance and altered sleep duration (frequent features of depression and anxiety) were significantly associated with elevated CRP and IL-6 (effect size for IL-6: 0.20; CRP: 0.12), supporting the strong inflammatory component of neurocognitive and mood dysregulation [13].

Emerging disease-specific studies reinforce this link across distinct chronic inflammatory conditions. Compared with controls, patients with fibromyalgia, a condition marked by widespread pain and fatigue, presented significantly elevated levels of CRP and IL-6, which correlated positively with measures of pain sensitivity and emotional distress [14]. The study included 125 individuals with fibromyalgia and 30 pain-free controls whose diagnoses were based on the 2016 ACR criteria. High-sensitivity CRP levels were significantly greater in the fibromyalgia group than in the control group (ANOVA: F(1,106) = 8.802, *p* < 0.001). Higher hsCRP levels were positively correlated with greater somatic symptom burden, as measured by the Somatic Symptom Scale-8 (r = 0.287, *p* = 0.008), and with increased tender point counts (r = 0.307, *p* = 0.005), suggesting a link between low-grade systemic inflammation and heightened pain sensitivity. While BMI was also significantly associated with hsCRP levels, inflammatory markers remained marginally elevated in fibromyalgia patients even after controlling for BMI and other covariates (*p* = 0.052). These findings support the role of inflammation, particularly the CRP–IL-6 axis, in the pathophysiology of fibromyalgia and its related symptoms and provide further evidence for the concept of inflammation-driven central sensitization and its potential influence on both pain and emotional distress.

CRP expression was also analyzed in a well-characterized cohort of IBD patients, where multivariable regression models (to control for disease activity and medication use) demonstrated that elevated serum CRP correlated with higher scores on validated psychological assessment tools, including the Hospital Anxiety and Depression Scale (HADS). These findings suggest that systemic inflammation, which is mediated via the IL-6–CRP signaling pathway, may influence the gut–brain axis, contributing to the neuropsychological burden in IBD, with intestinal inflammation associated with comorbid anxiety and depressive symptoms. These data support the concept of CRP as a potential mechanistic partner or intermediary between peripheral immune activation and central affective dysregulation [15]. IL-6 and CRP are likely to play key roles in the crosstalk between peripheral inflammation and neuropsychiatric disorders. The upregulation of these genes not only mirrors systemic disease activity but also appears to be intricately involved in the pathogenesis of depression and anxiety, suggesting promising options for biomarker-guided interventions and targeted anti-inflammatory therapies.

### Integrative Focus and Scope of the Review

Here we explicitly describe the IL-6/CRP–mCRP axis as the mechanistic spine that links systemic inflammation with pain hypersensitivity, stress responsivity, and neurocognitive vulnerability. In the pages that follow, we will prioritize evidence that clarifies causal pathways from IL-6–driven hepatocyte CRP production and context-dependent mCRP generation (at activated vascular and immune surfaces) to endothelial activation, BBB vulnerability, and central sensitization. By contrast, broader topics (e.g., multi-omic correlates, psychiatric comorbidity spectra) are treated selectively when they provide supportive evidence how this axis amplifies pain and stress signals into neurodegenerative risk. This focus ensures that downstream sections, on pain biology, HPA feedback, BBB integrity, and therapeutic levers, cohere around a single, testable model, that being inflammation → IL-6/CRP → mCRP effector signaling → vascular–neuroimmune dysregulation → cognitive decline.

## 3. CRP/IL-6: Relationships Between Pain, Pain Perception and Sensitivity, and Inflammation

Interestingly, in a thorough analysis of all relevant studies that reported associations between pain and inflammation among individuals with an inflammatory-related chronic pain condition, only approximately half of the reviewed studies reported a significant association between pain symptomatology and inflammatory markers, whereas in some studies, the magnitude of proinflammatory cytokine expression remained high even after effective pain relief medication [16].

One seminal study examined the links between pain, inflammation, and psychosocial stress, and here, researchers investigated older adults who were providing care for a spouse with dementia and compared them to age- and socioeconomic-status–matched noncaregiving controls [17]. The use of standardized pain questionnaires and high-sensitivity assays to quantify circulating CRP levels, revealed that among the caregiving group, higher self-reported pain scores were significantly associated with elevated CRP concentrations. In contrast, this association was not observed in the matched control group. These findings suggest that chronic psychosocial stress, in this case, stress related to caregiving, may amplify the link between nociceptive input and systemic inflammatory responses. This study thus supports the hypothesis that stress exposure acts as a sensitizing factor, potentially enhancing inflammatory signaling pathways involved in pain processing and contributing to a proinflammatory state in vulnerable individuals.

**Figure 1:**
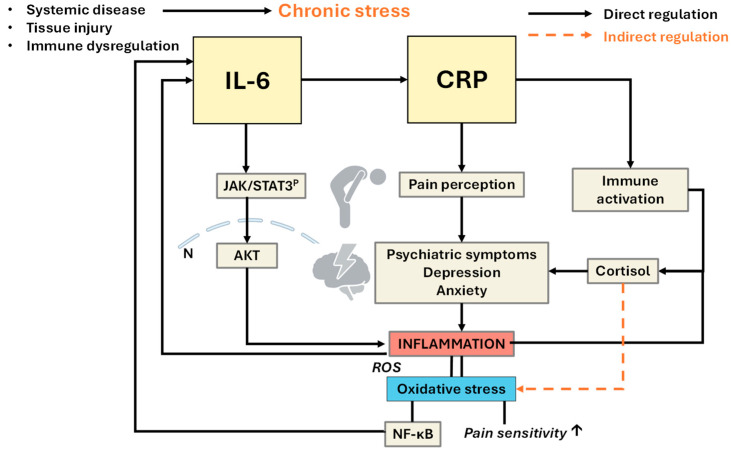
Proposed mechanistic pathway linking chronic inflammation, IL-6/CRP signaling, the stress response, and pain perception with neuropsychiatric and prognostic outcomes. Legend: This flowchart illustrates the signaling associations and hierarchies among chronic inflammation, immune signaling intermediates, neuroendocrine stress responses, and pain sensitivity. Chronic peripheral inflammation, initiated by systemic disease, tissue injury, or immune dysregulation, leads to the upregulation of proinflammatory cytokines, specifically interleukin-6 (IL-6). IL-6 plays a central role in activating the hepatic acute-phase response, resulting in the synthesis and release of C-reactive protein (CRP) by hepatocytes. Elevated CRP levels not only serve as a systemic marker of inflammation but also may contribute to immune cell recruitment and further cytokine activation. Simultaneously, chronic inflammation and immune activation stimulate the hypothalamic–pituitary–adrenal (HPA) axis, triggering the stress response. This leads to increased secretion of cortisol, a glucocorticoid hormone that modulates both immune and central nervous system functions. Although cortisol has anti-inflammatory properties, prolonged or dysregulated HPA activation may result in glucocorticoid resistance, thereby exacerbating inflammation and impairing its ability to suppress excessive oxidative stress and impair feedback control. IL-6, CRP, and cortisol collectively influence central pain processing pathways, enhancing pain sensitivity through oxidative stress and subsequent feedback of NF-κB to generate a stronger immune and proinflammatory environment.

The relative significance of the CRP–IL-6 axis in musculoskeletal pain was also highlighted by a systematic review conducted by van den Berg et al. (2018) [18], which analyzed data from ten previously published studies focusing on individuals with nonspecific lower back pain [18]. Here, the results revealed a consistent and statistically significant association between elevated levels of CRP and IL-6 and the presence or severity of nonspecific lower back pain. Interestingly, this pattern did not extend to other commonly measured proinflammatory cytokines, such as tumor necrosis factor-alpha (TNF-α), which showed no reliable correlation with pain outcomes across the studies reviewed. These findings suggest that CRP and IL-6 may serve as more robust peripheral biomarkers for inflammatory contributions to chronic musculoskeletal pain, particularly in the absence of identifiable structural abnormalities, as detailed in Figure 1.

Elevated levels of IL-6 and CRP have been shown to increase pain sensitivity and alter pain perception through effects on nociceptive neurons and glial activation in the spinal cord and brain. Additionally, chronic psychosocial stress, such as that experienced by caregivers or individuals with mood disorders, can act as a sensitizing factor, amplifying the relationship between inflammation and pain [19]. These interconnected signaling pathways contribute to a feedforward loop that sustains both peripheral inflammation and central sensitization. Over time, this is demonstrated to result in increased vulnerability to psychiatric symptoms (e.g., depression, anxiety, and fatigue) and influences prognostic outcomes, including disease severity, quality of life, and treatment responsiveness in conditions such as fibromyalgia, inflammatory bowel disease, and chronic low back pain.

In a systematic review by Dainese et al. (2023) [20], the authors examined the associations between markers of knee inflammation and altered pain perception mechanisms in individuals with knee osteoarthritis (OA). The review included nine studies with a combined total of 1889 participants, using robust databases (MEDLINE, EMBASE, Web of Science, and Scopus, searched up to 13 December 2022) and PRISMA methodology, with methodological quality rated via the National Heart, Lung, and Blood Institute tool. Pain perception was assessed via quantitative sensory testing (QST), pressure pain thresholds (PPTs) and temporal summation, and inflammatory status was determined through imaging markers (effusion, synovitis, and bone marrow lesions) and serum cytokine levels. The review revealed consistent associations between greater effusion/synovitis and lower PPTs, as well as an increased likelihood of neuropathic-like pain. Importantly, among the systemic markers assessed, higher serum CRP levels were positively associated with increased pain sensitivity, specifically lower PPTs and the presence of temporal summation, which is a sign of central sensitization. However, evidence linking CRP and pain was considered suggestive rather than conclusive because of the heterogeneity and limited number of high-quality studies identified. The authors hypothesized that inflammatory cytokines, including CRP, may play a role in sensitizing nociceptive pathways, although more longitudinal data are needed to strengthen causality [20].

A case–control study of patients with myofascial pain syndrome (MPS) compared 45 patients with matched healthy controls and measured a panel of inflammatory and oxidative stress biomarkers, including hs-CRP, phospholipase A2 (PLA2), malondialdehyde (MDA), total antioxidant capacity (TAC), and superoxide dismutase (SOD). Compared with the control group, the MPS group presented significantly greater serum levels of hs-CRP, PLA2, and MDA and lower antioxidant defenses (TAC and SOD). Crucially, hs-CRP levels were positively and significantly correlated with multiple dimensions of pain intensity, resting pain, activity-related pain, and night pain, as were the pressure pain threshold and pain duration. These findings suggest that CRP associated with systemic inflammation may amplify pain perception, even when pain is localized to musculoskeletal tissues. Additionally, lower antioxidant capacity is associated with greater pain intensity and reduced quality of life, indicating a potentially synergistic role of oxidative stress in sensitizing nociceptive circuits [21].

Taken together, this evidence reinforces the hypothesis that CRP serves not only as a biomarker of systemic inflammation but also as a potential mediator of pain amplification through mechanisms involving central sensitization, neuroimmune activation, and impaired oxidative regulation (see Figure 1). However, in addition to the positive correlation between elevated CRP and increased pain sensitivity, the current evidence highlights the need for more longitudinal, mechanistic, and interventional research to determine whether targeting CRP-related pathways can modulate pain perception in chronic inflammatory conditions [22].

In support of this hypothesis, the Tromso large population study measured hs-CRP and experimental pain sensitivity through cold-pressor tolerance. Using data from more than 10,000 participants, cold-pressor tests in which individuals submerged their dominant hand in 3 °C circulating water for up to 106 s were performed, and the outcome was modeled via Cox proportional hazard models, where early withdrawal indicated lower pain tolerance. There was a significant inverse relationship between hs-CRP levels and cold-pressor tolerance. Individuals with elevated hs-CRP levels (>3 mg/L) were more likely to withdraw their hand earlier than those with lower levels (≤3 mg/L), indicating reduced pain tolerance and heightened pain sensitivity. This association held after adjusting for multiple covariates, including age, sex, BMI, education, smoking, alcohol use, statin therapy, emotional distress, and chronic pain status (adjusted HR = 1.22, 95% CI: 1.09–1.36, *p* < 0.001). Notably, the relationship remained significant even among participants without chronic pain, indicating that subclinical inflammation itself may independently modulate pain sensitivity.

Intuitively, the data indicate that CRP could represent a potential novel biomarker for heightened pain sensitivity, reinforcing the role of low-grade inflammation in influencing individual differences in pain perception and suggesting potential implications for understanding pain vulnerability in otherwise healthy populations.

### Psychosocial Stress, Pain, and Inflammatory Biomarkers: The Complex Role of IL-6 and CRP in Systemic Low-Grade Inflammation

Recent research has increasingly focused on the mechanistic links between psychosocial stress, negative affect, and systemic low-grade inflammation (SLI), particularly through inflammatory mediators and the main subjects of this review, namely, IL-6 and CRP. These markers have emerged as reliable indicators of how emotional distress and somatic symptoms intersect to contribute to chronic inflammatory states and psychological morbidity.

In a population-based study by Graham-Engeland et al. (2022) [23], the authors explored the interactive effects of negative affect and physical pain on peripheral inflammatory markers among midlife adults. Using self-reported pain intensity and interference, coupled with ecological momentary assessments of affect (collected five times daily over a week), they measured serum CRP and a composite cytokine panel [23]. Their findings revealed that CRP levels were significantly elevated only in individuals who experienced both high negative affect and high levels of pain, suggesting a synergistic interaction between emotional distress and somatic discomfort in increasing systemic inflammation. This finding supports the hypothesis that affective and sensory pathways may jointly influence the inflammatory tone, which could contribute to the development or perpetuation of chronic diseases involving pain and mood dysregulation.

Similarly, van der Feltz-Cornelis et al. (2020) [24] investigated the role of IL-6 and hsCRP in somatic symptom and related disorders (SSRDs), a group of conditions characterized by significant emotional distress related to physical symptoms. In a clinical sample of 241 patients, both IL-6 and hsCRP were significantly elevated, independent of comorbid chronic illnesses. Interestingly, neither general stress levels nor adverse childhood experiences (with the exception of sexual trauma) were strongly associated with these biomarkers, suggesting that SLI in SSRD is not merely a byproduct of other stressors or medical conditions but is potentially intrinsic to the disorder. Moreover, elevated CRP was once again linked to comorbid depression and high pain levels, reinforcing its role as a mediator between emotional and somatic symptomatology [24].

Discrimination between the involvement of IL-6 and CRP operating within this system was described in a study examining the relationships among perfectionistic cognitions, perceived stress, and low-grade inflammation in a sample of 248 Canadian young adults (mean age ≈ 23 years). Using a diathesis-stress framework, this study examined whether individual differences in perfectionism influence the levels of IL-6 and CRP and whether these relationships are moderated by stress. Participants completed validated questionnaires assessing perfectionistic cognitions and perceived stress and provided blood samples for biomarker analysis alongside measurements of body fat percentage. Multiple regression analyses revealed a key distinction: perfectionistic cognition was not significantly associated with IL-6 at any stress level. In contrast, elevated CRP levels are significantly predictive of high levels of perfectionistic thinking only in the context of elevated perceived stress, independent of confounding variables such as smoking, adiposity, and sex [25].

Hence, the differential sensitivity of CRP and IL-6 to psychosocial stressors highlights CRP as a more stress-reactive marker within the perfectionism–inflammation (and possibly other genetically regulated personality traits) pathway, potentially offering mechanistic insight into how cognitive personality traits may contribute to chronic inflammatory states. Together, these studies reveal a bidirectional relationship in which psychological stress and pain amplify systemic inflammation, which in turn may lower pain thresholds and exacerbate mood disturbances via neuroimmune signaling. IL-6 likely plays a pivotal role in initiating these responses through its impact on the HPA axis and central nervous system sensitization, whereas CRP acts as a downstream effector and potential feedback amplifier within this neuroinflammatory loop. These findings suggest that assessing and targeting IL-6 and CRP could provide therapeutic insight into stress-related inflammatory disorders, where psychological and somatic symptoms are interlinked.

## 4. CRP, IL-6, Stress, and the HPA Axis: Integrative Signaling Mechanisms Linking Inflammation and Systemic Dysregulation

IL-6 and CRP may play different roles within the neuroimmune system, with distinct regulatory roles tied to stress-related activation of the hypothalamic-pituitary-adrenal (HPA) axis and systemic inflammation. In the perfectionistic cognition study (described briefly earlier in this review), under high levels of perceived stress, elevated CRP, but not IL-6, was significantly associated with perfectionistic cognitions, even after controlling for body fat, smoking status, and sex. These findings suggest that CRP may be more responsive to cognitive-emotional stress loads, reflecting downstream inflammatory responses rather than direct upstream cytokine activation. CRP, which is primarily a liver-derived acute-phase reactant, is typically regulated by IL-6 signaling; however, this study demonstrated that IL-6 may not directly mediate all psychosocial stress-linked inflammatory states, particularly when the stressor is cognitive or affective in nature [25].

Mechanistically, IL-6 plays a central role in initiating acute phase responses via hepatic induction of CRP, especially under neuroendocrine influence. The HPA axis, in response to stress, elevates cortisol secretion, which, under normal circumstances, inhibits proinflammatory cytokines, including IL-6. However, genetic variability in glucocorticoid receptor sensitivity (e.g., NR3C1 polymorphisms) can impair this anti-inflammatory control, leading to heightened circulating IL-6 levels even under normal cortisol exposure. Walsh et al. (2019) demonstrated this in a large cohort, where individuals with glucocorticoid receptor polymorphisms (rs6198) presented exaggerated IL-6 responses linked to shorter sleep duration, another proxy of chronic stress, indicating an impaired negative feedback loop from the HPA axis on immune signaling [26]. In patients with neuropsychiatric disorders such as schizophrenia and bipolar disorder, a lower cortisol/CRP ratio (interpreted as dysregulated HPA-immune balance) is associated with worse cognitive performance and more severe symptomatology, particularly in patients with schizophrenia. This finding suggests that insufficient glucocorticoid-mediated immunoregulation may lead to prolonged or unresolved CRP elevations, contributing to systemic and possibly neuroinflammatory pathology [27].

## 5. Monomeric CRP May Encapsulate an Additional Mechanosensing Mechanism

The dissociation of pentameric C-reactive protein (CRP) into mCRP upon contact with lysophosphocholine in activated or damaged cells and tissues should also be considered. A recent study revealed a novel mechanosensing function of CRP, showing that pathophysiological shear stress, such as that observed in aortic stenosis, can induce its dissociation into mCRP, which has potent proinflammatory and prothrombotic effects. Using both in vitro shear models and in vivo mouse models, the authors demonstrated that mCRP forms at vascular sites exposed to high shear, contributing to endothelial and platelet activation via increased ICAM-1, P-selectin, and TGF-β expression [28]. Importantly, this work suggested that mCRP acts as a mechanoresponsive molecule. In the context of pain sensitivity and thresholds, these findings suggest a potential mechanistic pathway whereby mechanical forces and vascular stress could influence local or systemic inflammation via mCRP production. Given the role of mCRP in activating inflammatory pathways and sensitizing immune and endothelial cells, its shear-induced formation could contribute to heightened nociceptive sensitivity and lowered pain thresholds, especially in vascular or stress-related pain syndromes. This finding indicates a potential functional role of mCRP as a link between biomechanical stress and inflammatory pain amplification, but further investigation is needed to establish this hypothesis.

Further evidence supports the theory that mCRP plays a role in enhancing pain sensitivity and lowering pain thresholds, particularly in conditions involving vascular or biomechanical stress. Unlike its pentameric form, mCRP exhibits strong proinflammatory activity by activating endothelial cells, leukocytes, and platelets and upregulating adhesion molecules such as ICAM-1 and P-selectin. It also stimulates the release of cytokines, including IL-6 and TNF-α, which are known to sensitize nociceptors and amplify pain perception. Chronic pain conditions such as complex regional pain syndrome (CRPS) and OA demonstrate a link between systemic inflammation and central sensitization, a hyperreactive state of the central nervous system to pain. Although most clinical CRP measurements do not (or have not yet been able to distinguish between isoforms), elevated CRP levels in patients with OA have been correlated with increased pain sensitivity/lower pain thresholds, suggesting a key role for mCRP in this process [20]. Moreover, several studies have shown that mechanical shear stress, such as that observed in aortic stenosis, can cause the dissociation of pentameric CRP into its monomeric form, highlighting a potential mechanosensory pathway that links physical stress to inflammatory activation [28,29]. These findings imply that mCRP may act as a molecular bridge between biomechanical stress and inflammatory pain amplification, offering new insights into the pathophysiology of chronic pain and potential therapeutic targets.

**Figure 2:**
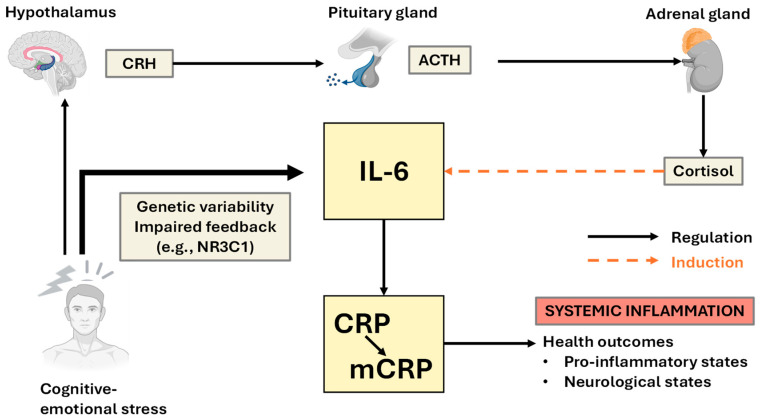
Mechanistic model linking psychological stress, IL-6-CRP, and systemic inflammation to health outcomes. Exposure to stressors activates the hypothalamus, leading to the release of corticotropin-releasing hormone (CRH) and subsequent activation of the pituitary–adrenal axis, resulting in cortisol production. Both central and peripheral stress responses induce IL-6 production, which stimulates the hepatic synthesis of CRP. Genetic variability and impaired feedback mechanisms (e.g., NR3C1) can further enhance IL-6 responses to cognitive-emotional stress. Elevated CRP, particularly in its monomeric form (mCRP), amplifies systemic inflammation, contributing to adverse health outcomes, including proinflammatory and neurological abnormalities. The diagram highlights regulatory (solid arrows) and inductive (dashed arrows) relationships among stress, neuroendocrine, and inflammatory mediators, illustrating how psychosocial and biological factors converge to influence inflammation-related disease risk and chronic pain sensitivity.

In summary, IL-6 is a key upstream cytokine in stress-induced immune activation, whereas CRP serves as a downstream surrogate for systemic inflammation. Stress-induced dysregulation of the HPA axis and altered glucocorticoid sensitivity can uncouple negative feedback mechanisms, resulting in sustained increases in IL-6 and CRP. This chronic proinflammatory state may underlie multiple stress-related physical and mental health outcomes, reinforcing the importance of psychoneuroendocrine balance in both resilience and disease susceptibility (see Figure 2).

### 5.1. CRP/mCRP-Associated Genetic and Epigenetic Considerations and AI Stratification of Risk

The IL-6–CRP axis is a critical mediator of inflammation operating at the intersection of mechanical, psychological, and genetic influences. Polymorphisms in the IL-6 gene, such as the -174G>C variant (rs1800795), have historically been shown to modulate transcriptional activity and are associated with heightened inflammatory responses to psychosocial stress and increased risk for CVD and depression [30]. Similarly, common CRP gene polymorphisms (e.g., rs1205 and rs3091244) influence baseline CRP levels and reactivity, contributing to interindividual variability in systemic inflammation, whereas variants in NR3C1 and BcLI, which encode the glucocorticoid receptor, affect HPA axis feedback and cortisol sensitivity, further modulating IL-6 and CRP expression in response to chronic stress and are also associated with increased glucocorticoid sensitivity, a greater insulin response, and, in some cases, greater abdominal obesity. In contrast, the ER22/23EK polymorphism is linked to GC resistance, a favorable metabolic profile, lower CRP levels, and improved survival. ER22/23EK carriers also presented a reduced risk of dementia and healthier brain aging [31].

Additionally, a study of 150 patients with MDD was conducted. Recent life events, but not childhood trauma, were associated with blunted glucocorticoid receptor signaling (lower SGK1 and FKBP5 expression postdexamethasone), elevated CRP and lymphocyte counts, and a reduced antidepressant response. Nearly 40% of patients experienced ≥3 recent severe events, which strongly predicted immune and HPA axis dysregulation. These findings indicate the distinct biological impact of proximal stressors in MDD and support the early integration of psychotherapy or immune-modulating treatments in affected individuals [32].

In addition to genetic variation, epigenetic mechanisms play a significant role in tuning the IL-6–CRP inflammatory response. Promoter methylation of the IL-6 and CRP genes has been linked to early-life adversity and chronic stress, with hypomethylation correlating with increased expression and elevated inflammatory tone [33]. Histone modifications and microRNAs, including miR-146a and miR-155, associated with CRP expression modulate NF-κB signaling and cytokine transcription, contributing to either homeostatic control or exaggerated inflammatory responses, for example, in diabetic patients with CAD [34]. Recently, mCRP itself was shown to directly promote histone H3 citrullination, a hallmark of neutrophil extracellular trap (NET) osis. This epigenetic alteration was shown to occur downstream of p38 MAPK signaling and NADPH oxidase activation, linking the conformational change in CRP directly to chromatin remodeling and innate immune activation. The authors further confirmed that blocking CRP dissociation reduced both NET formation and histone citrullination in ex vivo human whole blood models of trauma, highlighting mCRP as an inflammatory mediator capable of driving histone modification in neutrophils [35].

**Figure 3:**
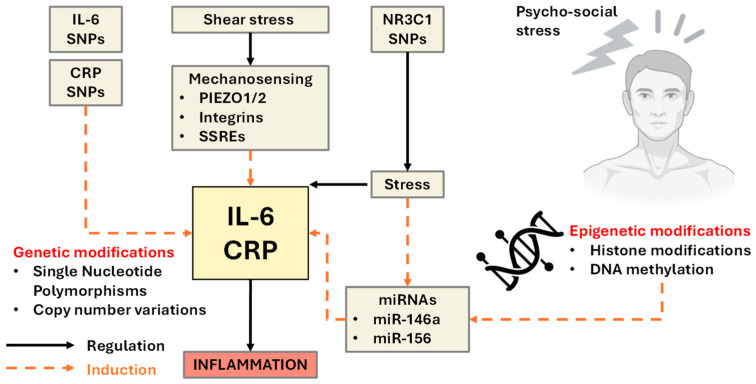
Genetic and epigenetic regulation of IL-6 and mCRP/CRP expression in response to stress and mechanical stimuli: This schematic illustrates the multifactorial regulation of interleukin-6 (IL-6) and C-reactive protein (CRP) expression, integrating genetic, epigenetic, mechanical, and psychological inputs. Single nucleotide polymorphisms (SNPs) in the *IL6* and *CRP* genes (left) modulate transcriptional responsiveness to internal and external stimuli. Mechanical forces such as shear stress activate mechanosensitive pathways (e.g., PIEZO1/2 ion channels, integrins, and shear stress–responsive elements [SSREs]) that induce IL-6 and CRP expression. Stress activates the hypothalamic–pituitary–adrenal (HPA) axis, and its regulation is influenced by genetic variants in *NR3C1* (glucocorticoid receptor gene), which in turn modulate the feedback of inflammatory cytokine expression. Epigenetic modifications, including histone acetylation and DNA methylation, further fine-tune the transcriptional landscape of the IL-6 and CRP genes and are influenced by early-life stress, environmental exposure, and chronic psychological load. MicroRNAs (e.g., miR-146a and miR-155) act as posttranscriptional regulators of the IL-6/CRP pathway and are also responsive to stress-induced epigenetic reprogramming. The convergence of these regulatory layers culminates in increased IL-6 and CRP expression, promoting systemic inflammation and contributing to stress-related pathologies. The solid arrows represent direct regulatory relationships; the dashed arrows indicate inductive or modulatory effects.

These regulatory layers are also responsive to mechanosensory stimuli, such as shear stress, via the activation of Piezo channels, integrins, and shear stress-responsive elements (SSREs) in endothelial cells. This mechanotransduction can induce the expression of IL-6 and CRP, particularly in vascular regions exposed to disturbed flow, implicating the axis in cardiovascular pathology and inflammation-associated neuropsychiatric conditions (Figure 3) [28].

### 5.2. Genetics, Epigenetics, and AI as Complementary Stratification Layers

Genetic variation (e.g., IL6, CRP, NR3C1), stress-linked epigenetic marking, and machine-learning risk models are best viewed here as adjunct amplifiers rather than alternative mechanisms. Their value is to identify individuals with heightened inflammatory tone or impaired HPA feedback, and to flag those biased toward pCRP → mCRP conversion at vascular interfaces. They may also operationalize multimodal data (biomarkers, QST, imaging, stress physiology) into actionable risk tiers. Therefore, omics and AI whilst not replacing the mechanistic core, may refine who is most susceptible to the IL-6/CRP–mCRP cascade and when to intervene. Future work should therefore report isoform-aware metrics (absolute mCRP and mCRP/CRP ratio) alongside genetic/epigenetic panels and add these features in pre-registered predictive models with external validation, so that stratification directly serves mechanism-based prevention or treatment trials.

## 6. mCRP Pathways to Neurodegeneration: Pathways to BBB Disruption and Beyond

As stated earlier, increasing evidence implicates anxiety and chronic stress-related psychiatric disorders as significant, modifiable risk factors for cognitive decline and dementia. In a comprehensive meta-analysis of six prospective cohort studies including over 10,000 participants, Santabárbara et al. (2019) [36] reported that individuals with clinically significant anxiety had a 29% increased risk of developing dementia (RR = 1.29; 95% CI: 1.01–1.66), even after adjusting for age and other confounders. The authors could not distinguish whether anxiety was a prodromal symptom of dementia or a contributing causal factor [36]. Nevertheless, these findings and others support a robust temporal association between chronic affective stress states and subsequent neurodegeneration, prompting further exploration into the underlying biological mechanisms involved.

One such mechanism may involve disruption of the BBB, a critical interface regulating CNS immune privilege and neurovascular homeostasis. Analysis of a wide range of postmortem, biomarker, and neuroimaging studies in psychosis revealed consistent evidence of BBB dysfunction in schizophrenia and related disorders, with downstream effects including increased CNS exposure to peripheral inflammatory molecules, glutamatergic dysregulation, and synaptic dysfunction. Importantly, compromised BBB integrity has been shown to reduce the efficacy of antipsychotic drugs and contribute to treatment resistance [37].

**Figure 4:**
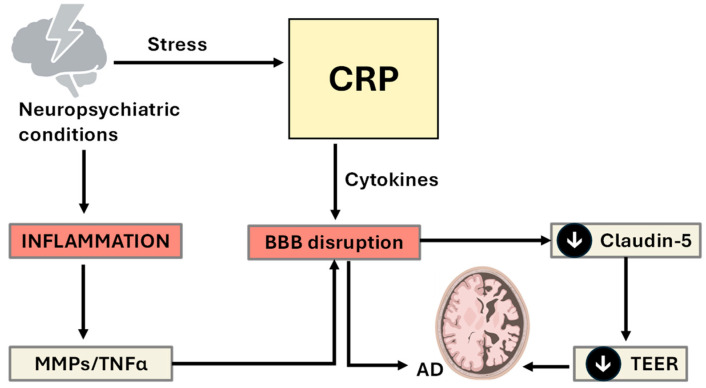
Pathways linking psychological stress to blood-brain barrier disruption and Alzheimer’s disease, highlighting molecular and cellular mediators. This schematic illustrates how chronic psychological stress and associated neuropsychiatric conditions (e.g., anxiety and depression) contribute to systemic inflammation and cerebrovascular dysfunction. Stress-induced upregulation of mCRP and proinflammatory cytokines leads to blood-brain-barrier (BBB) disruption, which is characterized by reduced tight junction protein expression (notably claudin-5) and increased permeability. Disruption is driven in part by elevated matrix metalloproteinases (MMPs) and tumor necrosis factor-alpha (TNF-α), which degrade endothelial integrity and exacerbate inflammatory signaling. Experimental evidence from iPSC-derived brain microvascular endothelial cells shows that decreased transepithelial/transendothelial electrical resistance (TEER) is a BBB-deficit subtype, that is mechanistically linked to MMP-1 and TNF-α activity. These alterations facilitate neuroimmune crosstalk and loss of vascular protection, thereby increasing the risk of Alzheimer’s disease (AD)-related pathology (down black arrows mean decrease).

In vitro findings using iPSC-derived brain microvascular endothelial cells (BMECs) as models of BBB characteristics in patients with schizophrenia and bipolar disorder demonstrated that while average BBB integrity appeared intact, a subset of BMECs derived from psychosis patients presented reduced transendothelial electrical resistance (TEER), increased permeability to small molecules, and decreased claudin-5 expression, which are hallmarks of BBB breakdown [38]. This BBB-deficit subgroup also presented elevated matrix metalloproteinase-1 (MMP1) activity, and functional improvements were observed with TNF-α and MMP1 inhibition, indicating inflammation-driven endothelial vulnerability (see Figure 4). Together, these studies provide some mechanistic evidence implicating chronic stress and psychiatric comorbidities (especially anxiety and psychosis), resulting in neurovascular dysfunction via inflammatory and endothelial pathways, disrupting the BBB and enabling neuroimmune crosstalk. This impaired barrier function may facilitate the entry of neurotoxic proteins, cytokines, and immune cells into the CNS, accelerating neurodegenerative processes associated with AD. A full characterization of these mechanisms and crosstalk should enable the identification of vulnerable subgroups, enabling stratified targeting of neuroinflammation and BBB stability as preventive strategies against dementia in high-risk psychiatric populations [39].

### Evidence Supports a Central Role for CRP/mCRP in Determining the ‘Risk’ of Conversion

In addition to its classical role as an inflammatory biomarker, CRP actively contributes to tissue pathology through complex signaling cascades. Studies have shown that CRP can impair cell regeneration and promote tissue fibrosis and inflammation via the CD32 receptor, leading to the activation of key proinflammatory and profibrotic pathways, including the NF-κB, TGF-β/Smad3, and mTOR signaling pathways (reviewed by [40]. For example, when CRP transgenic diabetic mice (CRPtg-db/db) expressing human CRP were compared with db/db diabetic controls, CRPtg-db/db mice developed significantly more severe T2DN, as evidenced by higher fasting blood glucose, increased microalbuminuria, and more pronounced renal inflammation and fibrosis. These phenotypic changes are associated with increased activation of CRP-CD32b signaling, alongside upregulation of the NF-κB, TGF-β/Smad3, and mTOR pathways in kidney tissue [41].

**Figure 5:**
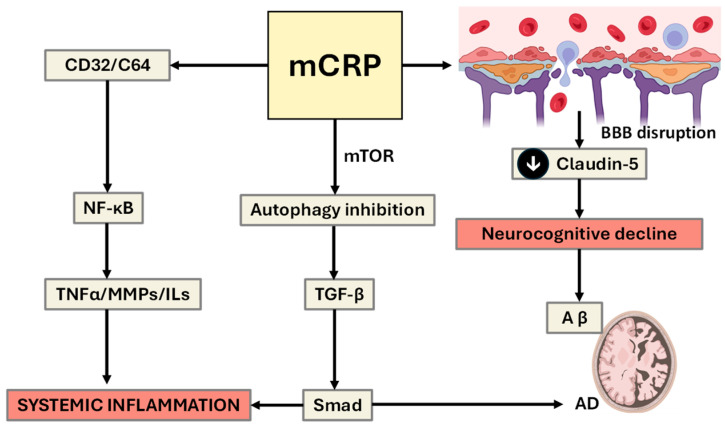
Signaling pathways linking CRP and mCRP to inflammation, BBB disruption, and neurodegeneration. This schematic illustrates how CRP and mCRP act as proinflammatory mediators through distinct but overlapping signaling cascades. Shear-induced mechanical stress promotes the dissociation of pentameric CRP (pCRP) into its monomeric form (mCRP), thereby linking vascular biomechanics with localized inflammatory signaling and endothelial activation. CRP activates CD32/CD64-mediated NF-κB-dependent proinflammatory signaling pathways, promoting systemic inflammation and tissue remodeling. Upon dissociation, mCRP exerts stronger pathogenic effects, including the inhibition of autophagy via mTOR signaling, the activation of TGF-β and, ultimately, SMAD-associated inflammation. mCRP also impairs endothelial homeostasis and contributes to blood-brain barrier (BBB) breakdown, partly through the downregulation of tight junction proteins such as claudin-5. The resulting BBB dysfunction facilitates neuroimmune activation, promotes amyloid-beta (Aβ) accumulation, and contributes to neurocognitive decline and Alzheimer’s disease (AD) pathology. The diagram highlights the central role of mCRP in linking chronic inflammation to neurodegenerative disease risk (down black arrow means decrease).

Mechanistically, CRP induced Smad3 phosphorylation through both early direct activation (within 15 min) via CD32b–ERK/p38 MAPK signaling and delayed activation (24 h) via a TGF-β1-dependent pathway. Furthermore, CRP activated mTOR signaling at 30 min, which was shown to be Smad3 dependent; Smad3 was found to physically bind to mTOR, and this interaction was functionally significant, as both a neutralizing antibody against CD32b and a specific Smad3 inhibitor abolished CRP-induced mTOR activation. Finally, the authors demonstrated that CRP promoted renal fibrosis by increasing CTGF and collagen I expression, an effect that was blocked by the mTOR inhibitor rapamycin, confirming the critical role of the CD32b–Smad3–mTOR axis. These findings establish CRP not only as a marker but also as a pathogenic effector in T2DN, mediating both inflammatory and fibrotic responses through distinct signaling pathways. These same pathways have been implicated in BBB disruption, particularly under diabetic and inflammatory conditions. The activation of mTOR and NF-κB suppresses autophagic clearance, a process essential for maintaining endothelial cell health and tight junction integrity. Notably, CRP downregulates claudin-5, a critical component of BBB tight junctions, whereas Smad3 and mTOR activation further destabilizes endothelial function [42]. These mechanisms suggest a direct role for CRP in facilitating the entry of inflammatory mediators and neurotoxic proteins into the central nervous system, potentially linking chronic systemic inflammation to neurodegenerative disease progression (Figure 5).

This pathophysiological cascade becomes especially relevant in the context of chronic pain and stress, which are independently associated with both elevated CRP levels and AD-related biomarker expression. Bell et al. (2024) [43] demonstrated that in older men, chronic pain was associated with significantly elevated plasma amyloid-beta (Aβ42, Aβ40) levels, and this relationship was amplified when high-sensitivity CRP (hs-CRP) was also elevated. The interaction between systemic inflammation and the amyloid load corresponds to a reduced hippocampal volume, a hallmark of early AD [43]. Other similar studies reported that systemic inflammation (as measured by hs-CRP and IL-6) and chronic stress (measured via hair cortisol measurements) were associated with subtle reductions in cortical thickness in healthy middle-aged adults [44]. Together, these studies support a mechanistic link between pain, reduced pain thresholds, CRP-mediated inflammation, BBB permeability, and neurodegenerative vulnerability, wherein CRP acts both as a marker and a driver of inflammation-induced cognitive decline.

## 7. Modifying Pain Perception and Thresholds: Therapeutics and Protective Solutions

**Figure 6:**
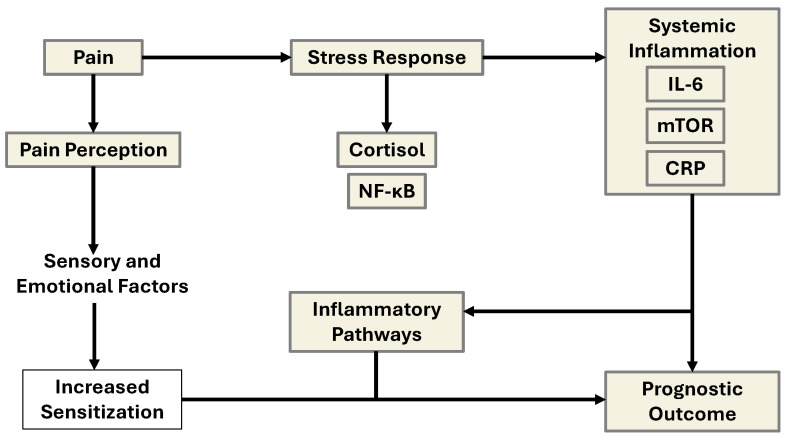
Schematic representation of the interrelated mechanisms linking pain, stress, inflammation, and prognostic outcomes via IL-6 and mCRP/CRP signaling. This diagram illustrates the interconnected biological pathways through which chronic pain and emotional distress contribute to systemic inflammation and poor clinical outcomes. Pain, modulated by both peripheral nociceptive input and central sensory-emotional processing, leads to altered pain perception. This altered perception, especially under the influence of chronic psychosocial stress, activates the hypothalamic–pituitary–adrenal (HPA) axis, triggering a stress response characterized by cortisol release. Elevated cortisol and subsequent activation of nuclear factor kappa-light-chain-enhancer of activated B cells (NF-κB) lead to increased transcription of proinflammatory genes. This cascade promotes the release of systemic inflammatory mediators, including interleukin-6 (IL-6) and C-reactive protein (CRP)**,** with mTOR signaling acting as a metabolic and growth regulator under inflammatory stress. The elevated inflammatory burden feeds back to sensitize pain pathways (via glial activation and peripheral sensitization), further exacerbating pain perception and emotional distress. Over time, this cycle contributes to maladaptive inflammatory pathways, which worsen overall prognostic outcomes by promoting disease progression, impaired healing, or psychiatric comorbidity.

The pain threshold refers to the minimal stimulus intensity required to elicit a pain sensation and is shaped by a variety of biological and physiological factors. Peripheral determinants include the density and excitability of nociceptors, which may be lowered by inflammation, injury, or sensitizing mediators such as prostaglandins and bradykinin. Genetic polymorphisms, particularly in genes such as SCN9A, catechol-O-methyltransferase (COMT), and OPRM1, can influence nociceptive signaling, whereas hormonal fluctuations and neurological integrity further modulate threshold sensitivity. Endogenous opioid systems (e.g., endorphins and enkephalins) and demographic factors such as age and sex also play roles in threshold variability. In contrast, pain perception encompasses the subjective and emotional response to painful stimuli, shaped primarily by central nervous system processing in areas such as the thalamus, insula, and anterior cingulate cortex. Psychological states (e.g., anxiety, depression, catastrophizing), previous pain experiences, and social context significantly influence how pain is experienced. Moreover, inflammatory mediators such as IL-6, TNF-α, and CRP have been implicated in enhancing pain sensitivity and perception, particularly in chronic pain conditions, highlighting the complex interactions among biological, psychological, and environmental modulators in shaping the overall pain experience [45] (Figure 6).

The model emphasizes that both psychological and immunological interventions targeting IL-6/CRP signaling or stress regulation could disrupt this loop and improve long-term health outcomes. Specifically, chronic pain often involves a reduction in the pain threshold due to peripheral sensitization to inflammatory agents such as prostaglandins, bradykinin, and cytokines such as IL-6 and TNF-α, which in turn enhance nociceptive transmission and disrupt homeostatic pain inhibition [46,47]. Central sensitization and persistent systemic inflammation have also been mechanistically linked to neurodegeneration and AD. A recent study by Bell et al. (2024) [43] demonstrated that older adults experiencing chronic pain presented elevated plasma amyloid-β (Aβ42 and Aβ40) levels, especially in the presence of high CRP, which exacerbates low-grade systemic inflammation. These elevations are significantly associated with hippocampal atrophy, suggesting a convergence between pain, inflammation, and AD pathology [43]. The synergistic interaction between pain and CRP may facilitate a neurotoxic environment, increasing the amyloid burden and impairing synaptic integrity.

Genetic predisposition further modulates pain sensitivity and susceptibility to cognitive decline. Polymorphisms in SCN9A (encoding Nav1.7 sodium channels), COMT (affecting catecholamine degradation), and OPRM1 (μ-opioid receptor) have been linked to altered nociceptive signaling and pain perception. Importantly, these genes also interact with stress and inflammatory pathways, which may amplify neurodegenerative risk [48,49]. The presence of the APOE ε4 allele, which is already the strongest known genetic risk factor for AD, may further potentiate the effects of inflammation on neurovascular integrity and amyloid deposition, especially under chronic stress- or pain-related conditions. Endocrine and neuroimmune responses are also key intermediaries. Elevated hair cortisol concentrations, an index of prolonged HPA axis activation, have been associated with both heightened pain perception and cortical thinning in middle-aged individuals [50]. Similarly, reductions in heart rate variability (HRV), reflecting autonomic imbalance and stress sensitization, have been observed in both chronic pain sufferers and individuals at risk for cognitive impairment. These stress-linked biomarkers interact with inflammatory cytokines to increase BBB permeability and microglial activation, which are the major hallmarks of early AD pathology.

**Figure 7:**
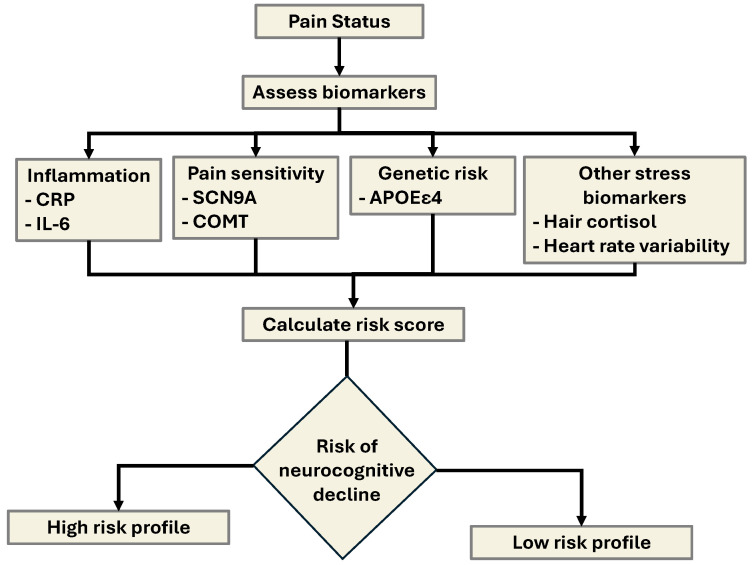
Algorithm framework for predicting neurocognitive decline in individuals with chronic pain via multimodal biomarkers. This flowchart outlines a predictive model integrating biological, physiological, and psychological inputs to assess the risk of Alzheimer’s disease (AD) or related neurocognitive decline. The algorithm accepts features from six key domains: (1) biomarkers, including inflammatory (hs-CRP, IL-6), neurodegenerative (Aβ42/40, NfL, tau), and genetic markers (SCN9A, COMT, OPRM1 SNPs); (2) neuroimaging metrics, such as hippocampal volume and cortical thickness; (3) pain sensitivity data from quantitative sensory testing (QST) and threshold scores; (4) psychological assessments, including measures of anxiety, depression, and catastrophizing; (5) stress physiology, including hair cortisol and heart rate variability (HRV); and (6) demographics, particularly age, sex, and APOE genotype. These inputs are processed through a machine learning model (e.g., logistic regression, random forest, or deep learning) to yield a personalized risk estimate of neurocognitive decline. The model is designed to support early identification and stratification of high-risk individuals for targeted prevention or intervention strategies utilizing the features and biomarkers shown in Table 1 below.

Finally, fluid biomarkers of neurodegeneration, including neurofilament light chain (NfL) and total tau (t-tau), are gaining traction as dynamic indicators of neuronal injury in both pain and dementia contexts. The presence of high NfL in conjunction with systemic inflammation may serve as a useful predictor of declining neurocognitive function, particularly in aging populations with comorbid pain syndromes. Hence, a multidimensional model in which chronic pain and s biological correlates, particularly systemic inflammation (CRP, IL-6, TNF-α), genetic variants (SCN9A, COMT, APOE ε4), and neuroendocrine stress biomarkers (HCC, HRV), may serve as early predictors of neurodegenerative risk. Integrating these biomarkers into predictive algorithms (as the example flow chart in Figure 7 shows) could aid in the early identification of individuals most vulnerable to AD, especially within the growing population living with chronic pain. Future longitudinal studies are warranted to validate biomarker-based risk stratification and to guide targeted interventions aimed at modifying this trajectory.

AI-component risk framework:

We propose a pragmatic, risk stratification framework that treats the IL-6 → CRP → mCRP cascade as the mechanistic core linking systemic inflammation with pain hypersensitivity, stress responsivity, and neurocognitive vulnerability. At first assessment, the model would estimate 12-month risk of (i) persistent high pain and functional impairment, (ii) clinically significant depressive/PTSD symptom burden, and (iii) cognitive decline. Candidate inputs prioritize biology that is both measurable and modifiable: hs CRP, mCRP and the mCRP/CRP ratio, IL-6 (±TNF-α), neural injury/astroglial markers (NfL, GFAP), AM cortisol and diurnal slope, simple wearables-derived sleep/HRV summaries, and, when available, kynurenine pathway indices (KYN/Trp, KYNA/QA). Brief psychometrics (pain interference, PHQ-9, GAD-7, PCL-5), cognition screens (e.g., MoCA), and context covariates (age, sex/menopausal status, BMI, comorbidities, medications, activity, smoking, socioeconomic factors) complete a clinically tractable feature set. Genetic/epigenetic markers (e.g., IL6, CRP, NR3C1, FKBP5, APOE) are positioned as optional refiners to identify individuals biased toward heightened inflammatory tone or pCRP→mCRP conversion.

**Table 1:** Key biological, physiological, and psychological markers associated with increased risk of Alzheimer’s disease (AD), particularly in the context of pain sensitivity and inflammation. The table outlines a multidomain risk profile including inflammatory biomarkers (e.g., hs-CRP, IL-6), neurodegenerative indicators (e.g., Aβ42/40, tau, NfL), pain processing measures (e.g., QST, pain thresholds), genetic polymorphisms (e.g., SCN9A, COMT, OPRM1), stress physiology markers (e.g., hair cortisol, HRV), and neuroimaging traits (e.g., hippocampal volume, cortical thickness). Psychological factors and demographics further contribute to risk stratification and individual variability in vulnerability.

Feature Type	Key Biomarkers
Biomarker Panel	hs-CRP, IL-6, Aβ42/40, NfL, tau, SCN9A/COMT/OPRM1 SNPs
Neuroimaging Metrics	Hippocampal volume, cortical thickness (optional)
Pain Metrics	Quantitative sensory testing (QST), pain threshold scores
Psychological Assessments	Anxiety, depression, catastrophizing scales
Stress Physiology	Hair cortisol, heart rate variability (HRV)
Demographics	Age, sex, APOE genotype

Structurally, the approach favors transparent, validated models: a regularized logistic (or Cox) baseline for interpretability, complemented by gradient-boosted trees to capture nonlinearity and interactions; mixed time-series and text can be reduced to summary features and fused late. Performance should be demonstrated with temporal validation, calibration (Brier/reliability), AUPRC alongside AUROC given class imbalance, and decision-curve analysis to show net clinical benefit; external, sex-disaggregated testing is required. Outputs are delivered as risk tiers (low/intermediate/high) mapped to pre-specified actions, enabling mechanism-matched care: for example, escalation to anti-inflammatory strategies when mCRP/CRP and IL-6 dominate, or neuromodulatory/sleep-focused interventions when HRV/sleep and affective measures drive risk. Re-estimation after 4–8 weeks supports response monitoring and adaptive care. In summary, the model operationalizes the inflammation–pain–neurocognition axis into a practical, clinically interpretable tool that strengthens translational relevance without diluting mechanistic focus.

A simple hypothetical example could be: A 65-year-old individual with chronic pain, elevated hs-CRP and IL-6, high Aβ42/40, low HRV, and SCN9A/COMT variants would have a high predicted risk of neurocognitive decline and AD. A detailed analysis of the potential therapeutic approach is beyond the scope of this review, but in summary, on the basis of the phenotype of elevated inflammatory markers CRP and IL-6, along with altered pain perception and autonomic dysfunction (e.g., low heart rate variability), predisposing a vulnerability in older adults with chronic pain, the proposal would be as follows: precision-tailored interventions aimed at reducing the inflammatory load and modulating stress physiology indicate that therapies targeting IL-6 signaling (e.g., IL-6 receptor antagonists) and central sensitization (e.g., gabapentinoids, duloxetine, or low-dose naltrexone) may help mitigate pain while dampening neuroinflammation and its downstream effects on brain structure and function [50].

Pharmacologic interventions could target IL-6 as the principal hepatic driver of CRP synthesis. Monoclonal antibodies such as tocilizumab and sarilumab, which inhibit IL-6 receptor signaling, markedly reduce circulating CRP levels and have shown cognitive and fatigue benefits in inflammatory disorders including rheumatoid arthritis and post-COVID syndromes. Recent data also suggest that chronic IL-6 blockade can indirectly limit peripheral mCRP formation and endothelial activation by lowering substrate (pCRP) availability [51]. Small molecules that stabilize pentameric CRP and prevent its dissociation (e.g., phosphocholine analogues) have demonstrated reduced vascular inflammation in preclinical models [52]. In addition, novel compounds, including peptide inhibitors of mCRP–CD16 interaction and nanobody-based mCRP neutralizers are under investigation for limiting mCRP-driven microglial and endothelial activation [53].

In addition to pharmacological strategies, noninvasive interventions such as mindfulness-based stress reduction, biofeedback, and structured exercise programs may enhance autonomic balance and downregulate inflammatory pathways. Regular aerobic training and structured exercise programs (including HIIT) are associated with significant reductions in CRP, particularly in cardiometabolic risk groups [54].

Nutritional interventions, including the Mediterranean diet and omega-3 fatty acid supplementation, also show promise in reducing IL-6 levels and promoting neurocognitive health. Moreover, recent interest in GLP-1 receptor agonists (e.g., semaglutide) has highlighted their dual benefits in metabolic control and neuroprotection, with trials examining their effects on AD prevention.

Given the connection between pain sensitivity, inflammation, and cognitive decline, multidomain interventions personalized by genetic (e.g., SCN9A, COMT polymorphisms), biomarker, and neuroimaging data may represent the most effective paradigm for protecting vulnerable individuals from progressive neurodegeneration [55,56].

Quantifying baseline pCRP/mCRP ratios may help identify patients with disproportionate mCRP activity who are most likely to benefit from CRP-modulating therapies. Isoform-selective assays have become available: a validated mCRP ELISA has been applied in clinical cohorts, and recent translational studies have reported circulating mCRP measurements in patient populations. These tools can support isoform-resolved stratification in interventional trials [9].

### Mechanism-Anchored Therapeutic Framing

To maintain coherence with our mechanistic focus, we have interpreted interventions primarily through their impact on the IL-6/CRP–mCRP pathway focused upon upstream IL-6 blockade to reduce hepatic CRP drive, stabilization or neutralization strategies to limit mCRP effector signaling at activated endothelium/immune surfaces, and stress- and pain-modulating approaches (exercise, autonomic balance, cognitive therapies) that reduce the feed-forward coupling between nociception, HPA dysregulation, and inflammatory tone. This should help to clarify why a therapy should work in this system and how its success ought to be measured (e.g., shifts in mCRP/CRP ratio, BBB-relevant markers, and pain sensitivity metrics), aligning trial design with the biology we propose.

## 8. Limitations (mCRP Limited Studies, Etc.)

In constructing this narrative review, we were unable to fully explore several critical regulatory systems due to scope limitations. First, microglial activation and neuroinflammation are omitted despite their central role in mediating systemic-to-central immune communication. Activated microglia, which can be detected via fluid biomarkers such as sTREM2 or YKL-40 and via TSPO PET imaging, are key drivers of synaptic dysfunction, tau phosphorylation, and neuronal loss in AD [57]. The inclusion of these markers in future models would enhance our understanding of how peripheral IL-6/CRP signaling translates into central neurodegenerative processes. However, a detailed discussion of cell-specific neuroimmune interactions was beyond our current paper’s framework. Second, we only briefly mentioned BDNF, glutamate excitotoxicity, and oxidative stress/mitochondrial dysfunction, each of which significantly influences pain sensitivity (e.g., neuropathic pain), inflammation, and cognitive decline. For example, inflammation-driven reductions in BDNF compromise neuroplasticity and resilience to stress, whereas altered glutamate transporter function contributes to excitotoxic damage in the hippocampus, and concomitant overactivation of NMDA receptors contributes to both pain amplification and hippocampal damage. Chronic IL-6/CRP elevation also modulates oxidative stress and mitochondrial impairment, key mechanisms in neurodegenerative pathology [58]. Although these systems are essential to a holistic biopsycho-neuroimmune model, fully integrating these interdependent pathways exceeds the constraints of our review. Going forward, comprehensive characterization of pain and dementia risk models will require inclusion of these regulatory axes in both observational and interventional study designs.

A key limitation across the literature reviewed is that most studies quantify only total/hsCRP and do not discriminate the mCRP isoform, despite mCRP’s distinct, locally pro-inflammatory biology. As a result, many associations between “CRP” and pain, stress, or cognitive outcomes likely conflate systemic acute-phase load pCRP with effector activity (mCRP), obscuring the mechanism and prognostic precision. Future work should use conformation-specific assays for plasma mCRP with rigorous preanalytics and vesicle-bound fractions, and report both absolute mCRP and the mCRP/CRP ratio using, standardized methods across laboratories Establishing these standards is essential to confirm whether mCRP and the mCRP/CRP ratio offer clinically actionable risk stratification beyond hs-CRP.

mCRP is currently measured using conformation-specific immunoassays that recognize epitopes exposed only on the monomeric isoform or by ligand-binding systems minimizing cross-reactivity with native pCRP. However, assay standardization remains limited, and differences in antibody pairs, calibrators, and reporting units produce substantial variability across laboratories. Moreover, pre-analytic factors such as sample matrix, anticoagulant type, storage, and freeze–thaw cycles can promote artifactual pCRP→mCRP conversion or loss of vesicle-bound mCRP, confounding quantification. These analytical and pre-analytical inconsistencies restrict cross-study comparability of absolute mCRP levels. Therefore, use of isoform-selective assays [9,59] and normalization through the mCRP/CRP ratio is recommended to mitigate methodological bias and better capture CRP isoform dynamics.

## 9. Conclusions

Chronic pain, systemic inflammation, and neurocognitive decline are increasingly understood as interrelated processes, with CRP and IL-6 acting as key mediators that bridge peripheral nociceptive signaling and central neurodegenerative mechanisms. The synergistic effects of pain and inflammation contribute to reduced pain thresholds, altered central processing, and increased risk of hippocampal atrophy and AD pathology. IL-6 and CRP therefore predict increased pain sensitivity, emotional distress, and cognitive vulnerability. Both biomarkers serve not only as indicators of inflammatory tone but also as active participants in neuroimmune dysregulation, influencing nociceptive processing, HPA axis activity, and neurovascular integrity. Monomeric CRP, in particular, appears to amplify peripheral and central sensitization through its interactions with the CD32, NF-κB, and mTOR–Smad3 signaling pathways, which also contribute to BBB breakdown and neurodegenerative risk. Elevated CRP and IL-6 levels are consistently associated with stress-perception phenotypes, cognitive impairment, and progression to dementia in vulnerable individuals, particularly when combined with pain and emotional stress. These findings support a feed-forward model of inflammation-induced hypersensitivity, where psychological, genetic (e.g., NR3C1, SCN9A, COMT), and physiological markers interact to drive cognitive decline.

Given these multifactorial interactions, future research should adopt an integrated multidomain biomarker model that includes inflammatory markers (IL-6, hsCRP), genetic variants, neuroimaging (e.g., hippocampal volume, cortical thickness), stress physiology (e.g., hair cortisol, HRV), and measures of pain sensitivity (e.g., QST, pain thresholds). This model could be embedded in prospective, longitudinal clinical trials aimed at early identification and stratification of individuals at risk for neurodegeneration, especially in chronic pain or psychiatric populations. Ideally, trials should be designed as adaptive, biomarker-guided intervention studies, combining anti-inflammatory therapies (e.g., IL-6R antagonists), stress-modulation techniques (e.g., biofeedback, mindfulness), and cognitive outcomes (e.g., episodic memory, executive function) as endpoints. Such trials would validate predictive frameworks and inform personalized interventions to halt or reverse inflammation-driven cognitive decline.

By elucidating the molecular and physiological pathways involved in processes ranging from proinflammatory cytokine cascades and BBB disruption to stress-related neuroendocrine dysregulation, the importance of integrative biomarker-based approaches for early identification and intervention in at-risk populations can be appreciated. Targeting inflammation-driven pain amplification may thus increase risk and enable stratified mitigation/protection of long-term cognitive vulnerability.

## Data Availability

No new data were created or analyzed in this study. Data sharing is not applicable to this article.

## Abbreviations

AD	Alzheimer’s disease
Aβ	Amyloid-beta
BBB	Blood–brain barrier
BMEC	Brain microvascular endothelial cell
BMI	Body mass index
CNS	Central nervous system
COMT	Catechol-O-methyltransferase
CRH	Corticotropin-releasing hormone
CRP	C-reactive protein
CRP/hsCRP	C-reactive protein
CRPS	Complex regional pain syndrome
HADS	Hospital Anxiety and Depression Scale
HPA	Hypothalamic–pituitary–adrenal axis
IL-6	Interleukin-6
mCRP	Monomeric CRP
MDA	Malondialdehyde
MDD	Major depressive disorder
MMPs	Matrix metalloproteinases
MPS	Myofascial pain syndrome
MR	Mendelian randomization
NfL	Neurofilament light chain
OA	Osteoarthritis
PLA2	Phospholipase A2
PPTs	Pressure pain thresholds
PTSD	Posttraumatic stress disorder
QST	Quantitative sensory testing
SLI	Systemic low-grade inflammation
SNPs	Single nucleotide polymorphisms
SOD	Superoxide dismutase
SSREs	Shear stress-responsive elements
SSREs	Stress–responsive elements
TAC	Total antioxidant capacity
TEER	Transendothelial electrical resistance
TNF-α	Tumor necrosis factor-alpha
